# The effect of press-needle therapy on postoperative nausea and vomiting in patients undergoing bronchoscopy under general anesthesia in China: a quasi-experimental study

**DOI:** 10.3389/fmed.2026.1813642

**Published:** 2026-06-05

**Authors:** Kunying Li, Taomei Lian, Jiaozhen Lou, Xin Liu, Guangzhao Zhang, Zaigui Li, Jingcai Gao, Qinqin Wang

**Affiliations:** 1 Henan Provincial Chest Hospital, Respiratory Endoscopy Center, Zhengzhou University, Zhengzhou, China; 2 Departments of Tuberculosis and Integrated Traditional Chinese and Western Medicine, Henan Provincial Chest Hospital, Zhengzhou University, Zhengzhou, China; 3 Hainan Xubairui Biotechnology Co., Ltd., Haikou, China; 4 Department of Tuberculosis Internal Medicine, Henan Provincial Chest Hospital, Zhengzhou University, Zhengzhou, China; 5 Department of Thoracic Surgery, Henan Provincial Chest Hospital, Zhengzhou University, Zhengzhou, China; 6 Department of Traditional Chinese Medicine Rehabilitation, Henan Provincial Chest Hospital, Zhengzhou, China; 7 Department of Research, Henan Provincial Chest Hospital, Zhengzhou University, Zhengzhou, China; 8 Department of Clinical Laboratory, Zhengzhou Daqiao Hospital, Zhengzhou, China

**Keywords:** bronchoscopy, general anesthesia, postoperative nausea and vomiting, press-needle therapy, quasi-experimental study

## Abstract

**Background:**

General anesthesia is commonly used in bronchoscopy, but postoperative nausea and vomiting (PONV) remain common and lack well-established prophylactic interventions.

**Objectives:**

To evaluate the efficacy of press-needle therapy in preventing PONV in patients undergoing bronchoscopy under general anesthesia in China.

**Methods:**

In this prospective quasi-experimental study, 216 patients scheduled for elective bronchoscopy under general anesthesia at Henan Provincial Chest Hospital were consecutively enrolled between September 2022 and December 2024. Participants were assigned by enrollment time to a press-needle group (PC6, LI4, ST36, applied 30 min pre-anesthesia and retained for 48 h) or a control group (standard care). Primary outcomes were nausea/vomiting rates and severity at four intervals (0–6 to 24–48 h post-op). Secondary outcomes were rescue antiemetic use and vital signs at baseline, immediately post-procedure, 6 and 24 h.

**Results:**

Generalized estimating equation analysis demonstrated that both groups experienced a significant reduction in the rates of nausea and vomiting over time (both *P* < 0.001). The press-needle group had significantly lower rates of nausea (OR = 0.417, 95% CI: 0.193–0.900, *P* = 0.026) and vomiting (OR = 0.355, 95% CI: 0.137–0.919, *P* = 0.033) than the control group. Furthermore, the press-needle group had significantly fewer patients experiencing mild, moderate, or severe nausea and vomiting (*P* < 0.001) than the control group. The overall use of rescue antiemetics was also significantly lower in the press-needle group [7 cases (6.48%) vs. 22 cases (20.37%), *P* = 0.004] than that in the control group. With respect to vital signs, systolic and diastolic blood pressures were significantly higher in the press-needle group immediately after the procedure (*P* < 0.001), but no significant differences were observed at 6 or 24 h postoperatively (*P* > 0.05).

**Conclusion:**

Press-needle therapy effectively reduces the rates and severity of PONV following bronchoscopy under general anesthesia, decreases the need for rescue antiemetic medication, and demonstrates a minimal impact on vital signs, suggesting its potential as a valuable adjunctive clinical intervention.

## Highlights

- Non-pharmacologic PONV prevention via press-needle therapy at PC6, LI4, ST36.
- Significantly reduced early (0–24 h) nausea and vomiting incidence post-bronchoscopy.
- Lowered rescue antiemetic use (6.48%) and symptom severity versus control (20.37%).
- Minimal, transient impact on vital signs, safe as adjunctive clinical intervention.
- First quasi-experimental evidence for this therapy in GA bronchoscopy patients.

## Introduction

Bronchoscopy under general anesthesia is a crucial procedure for the diagnosis and treatment of respiratory diseases. However, postoperative nausea and vomiting (PONV) represents one of its most common complications, with an incidence rate as high as 30%–50% (1, 2). PONV not only causes significant patient discomfort but also impedes postoperative recovery through multiple pathways: it can reduce appetite, cause sleep disturbances, and delay the initiation of physiotherapy, thereby hindering the rehabilitation process (3). Furthermore, PONV may lead to risks such as dehydration, electrolyte imbalances, and pulmonary aspiration of gastric contents (4). In severe cases, it can prolong hospital stays or increase readmission rates, consequently elevating healthcare costs (5, 6). Therefore, exploring effective and safe interventions for PONV is of great significance for improving patient experience and enhancing overall healthcare quality.

Currently, the clinical prevention and management of PONV primarily rely on pharmacological interventions, which encompass a variety of drug classes. These mainly include 5-HT_3_ receptor antagonists, dopamine D_2_ receptor antagonists, NK_1_ receptor antagonists, corticosteroids, antihistamines, and anticholinergics (7–10). However, these medications are often associated with various adverse effects. For instance, 5-HT_3_ receptor antagonists may cause headaches and constipation, D_2_ receptor antagonists can lead to sedation and arrhythmias, while corticosteroids carry risks of hyperglycemia and impaired wound healing (7, 9, 10). Additionally, evidence regarding the adverse effects of newer classes, such as NK_1_ receptor antagonists, remains insufficient, with preliminary reports including dizziness and headache (11). Consequently, while pursuing effective PONV prophylaxis, exploring interventions with fewer adverse effects and higher safety profiles holds significant clinical importance.

Press-needle therapy, a form of sustained acupoint stimulation, has seen increasing application in the field of postoperative rehabilitation in recent years. This therapy involves fixing fine press-needles on specific acupoints to provide continuous stimulation, thereby regulating physiological functions (12). The Neiguan (PC6) acupoint, a point on the Pericardium Meridian, is believed to soothe the chest, regulate qi, harmonize the stomach, and counteract rebellious qi (13). The Hegu (LI4) acupoint, the Yuan-Source point of the Large Intestine Meridian, functions to dispel wind, release the exterior, and unblock collaterals to relieve pain (14). The Zusanli (ST36) acupoint, the He-Sea point of the Stomach Meridian, can fortify the spleen, harmonize the stomach, and support vital energy (15). Modern research suggests that acupoint stimulation may exert antiemetic effects by modulating autonomic nervous system function and inhibiting the excitability of the central chemoreceptor trigger zone (16).

Despite the potential demonstrated by press-needle therapy in managing postoperative nausea and vomiting, high-quality clinical research focusing on its application in the specific context of bronchoscopy under general anesthesia remains relatively scarce. The confirmation of its efficacy and its broader clinical adoption urgently require more evidence-based medical support. Therefore, this study aims to evaluate the preventive effect of press-needle therapy on PONV following bronchoscopy and to investigate its impact on the usage rate of rescue antiemetics and patient vital signs, thereby providing a reliable basis for the standardized application of this technique in this patient population.

## Materials and methods

### Study design and population

This prospective quasi-experimental study consecutively enrolled patients who were scheduled to undergo bronchoscopy under general anesthesia, either as inpatients or outpatients in the Respiratory Department of Henan Provincial Chest Hospital, between September 2022 and November 2024. The inclusion criteria were as follows: (1) meeting the indications for bronchoscopy under general anesthesia as outlined in the Expert Consensus on the Clinical Application of Single-Use Bronchoscopy (17); (2) aged between 18 and 70 years; (3) American Society of Anesthesiologists (ASA) physical status classification I–III; and (4) voluntary participation with provision of written informed consent. Patients were excluded if they met any of the following criteria: (1) allergy to press-needles or presence of skin lesions/breakdown at the intended acupoint sites; (2) severe dysfunction of vital organs such as the heart, liver, or kidneys; (3) history of psychiatric disorders or cognitive impairment; or (4) use of antiemetic medication within 24 h prior to the procedure. This study was approved by the hospital’s Ethics Committee, and written informed consent was obtained from all participants.

### Intervention

Patients scheduled for bronchoscopy under general anesthesia were allocated into two groups based on their enrollment date: a press-needle group and a control group. Those enrolled between September 2022 and August 2023 were assigned to the control group, while those enrolled between September 2023 and November 2024 were assigned to the press-needle group.

Patients in press-needle group received press-needle therapy 30 min prior to the induction of anesthesia. The selected acupoints (bilaterally) were: Neiguan (PC6, located 2 cun proximal to the wrist crease, between the tendons of the palmaris longus and flexor carpi radialis), Hegu (LI4, on the dorsum of the hand, between the first and second metacarpal bones, at the midpoint of the radial side of the second metacarpal bone), and Zusanli (ST36, 3 cun below the Dubi (ST35), one finger-breadth lateral to the anterior crest of the tibia). Following routine skin disinfection, sterile press-needles (specification: 0.22 mm × 2.0 mm) were inserted perpendicularly into the acupoints and firmly secured with medical adhesive tape. The press-needles were retained for 48 h. Patients were instructed to avoid touching or pressing on the needle sites to prevent displacement.

Patients in control group received standard post-bronchoscopy care under general anesthesia, which included: Lying supine without a pillow for 6 hours postoperatively with the head turned to one side to prevent aspiration; Monitoring of vital signs (blood pressure, heart rate, respiratory rate, oxygen saturation) every 15 min for the first 30 min postoperatively, then every 30 min until 2 h postoperatively, and subsequently every hour once vital signs stabilized; Supplemental oxygen via nasal cannula (flow rate: 2–3 L/min) for 2 h postoperatively; Proactive communication with patients to alleviate anxiety, including explanations about potential postoperative nausea and vomiting to reduce psychological burden. No additional prophylactic antiemetic interventions (pharmacological or physical) were administered.

All patients underwent general anesthesia. Anesthesia was induced with propofol, fentanyl, and cisatracurium. Maintenance was achieved with continuous infusion of propofol and remifentanil, with titration based on the patient’s intraoperative vital signs. All bronchoscopic procedures were performed by experienced endoscopists, with the operative time controlled to approximately 30 min.

### Outcome

The primary outcomes were the rates of nausea and vomiting. The rates of nausea during different postoperative intervals were recorded for 0–6, 6–12, 12–24, and 24–48 after surgery. Nausea was defined as a subjective feeling of discomfort in the upper abdomen with an urge to vomit. The rates of vomiting during different postoperative intervals: Clinically assessed and recorded for the same time intervals. Vomiting was defined as the oral expulsion of gastric contents.

#### The secondary outcomes

(1) The severity of nausea and vomiting: The severity of nausea was graded according to the WHO criteria as: Mild (slight nausea, not interfering with daily activities), Moderate (distinct nausea, interfering with daily activities but no vomiting), or Severe (intense, unbearable nausea). The severity of vomiting was graded as: Mild (1–2 episodes), Moderate (3–4 episodes), or Severe (≥5 episodes).

(2) Use of rescue antiemetic medication: The number of patients requiring rescue antiemetics (e.g., ondansetron, dexamethasone) within 48 h postoperatively due to severe nausea or vomiting was recorded.

(3) Changes in vital signs: Systolic and diastolic blood pressure were monitored before anesthesia induction, immediately after the procedure, and at 6 and 24 h postoperatively.

### Covariates

Members of the study group uniformly trained the nursing staff involved in data collection. The patients’ data, including age, gender, weight, American Society of Anesthesiologists Physical Status Classification (ASA) score, type of examination, personal medical history, and smoking history. A uniform scale was filled for observation. The data were collected and imported into the computer after two specific researchers rechecked them to ensure accuracy.

### Sample size calculation

Sample size was calculated based on the primary outcome (incidence of postoperative nausea). According to a previous study (1), the incidence of PONV in patients undergoing bronchoscopy under general anesthesia was 56.6%. Assuming a clinically meaningful reduction in nausea incidence from 56.6% in the control group to 35% in the press-needle group (a relative reduction of approximately 38%), with a two-sided significance level (α) of 0.05 and statistical power (1−β) of 80%, the required sample size was calculated to be 80 patients per group using Pearson’s Chi-square test. Accounting for a 20% dropout rate, we aimed to enroll at least 100 patients per group. Thus, our final sample of 108 patients per group (total *N* = 216) exceeded this requirement.

### Statistical analysis

Statistical analysis was performed using SPSS software (version 27.0; IBM Corp., Armonk, NY, United States). Categorical data are presented as number and percentage (%), and between-group comparisons were performed using the chi-square test or Fisher’s exact test (when expected cell frequencies were <5). Continuous data are expressed as mean ± standard deviation (SD) after testing for normality using the Shapiro-Wilk test; normally distributed data were compared using the independent samples *t*-test, while non-normally distributed data were compared using the Mann-Whitney U test. For ordinal categorical data (e.g., severity grades of nausea and vomiting), the Mann-Whitney U test was used to compare the distributions between the two groups. For repeated measurements of outcomes across multiple time points (0–6, 6–12, 12–24, and 24–48 h postoperatively), a generalized estimating equation (GEE) model was employed to assess between-group effects, time effects, and group-by-time interaction effects. To account for multiple comparisons across the four postoperative time intervals, the Bonferroni correction was applied, with statistical significance set at *P* < 0.0125 for these specific comparisons. A two-sided *P*-value of less than 0.05 was considered statistically significant for all other analysis.

## Results

### Comparison of baseline characteristics between the two groups

**FIGURE 1:**
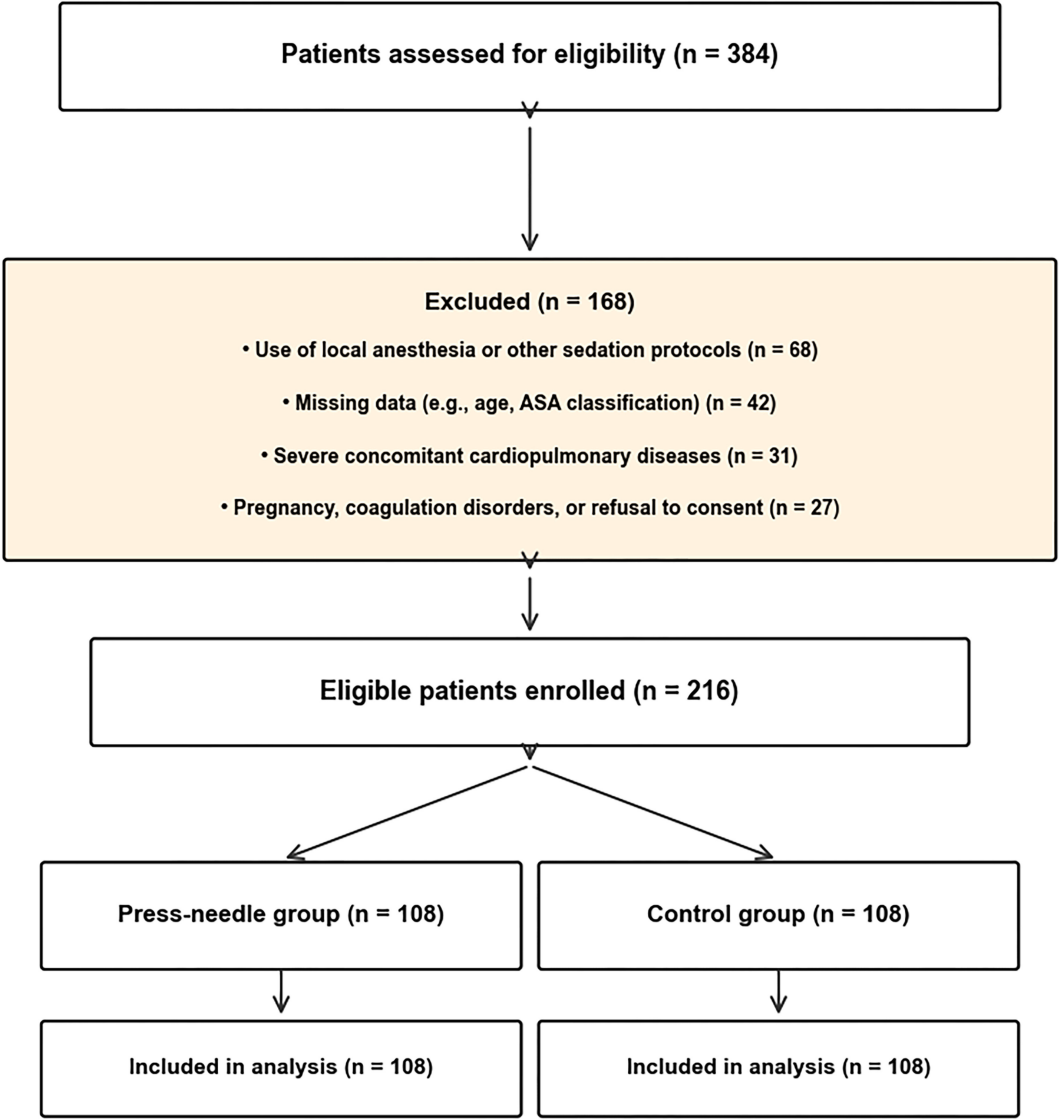
Flowchart of patient enrollment and exclusion.

A total of 384 patients scheduled for bronchoscopy under general anesthesia were initially assessed. Of these, 68 were excluded due to the use of local anesthesia or other sedation protocols, 42 were excluded for missing data (e.g., age, ASA classification), 31 were excluded due to severe concomitant cardiopulmonary diseases (such as acute decompensated heart failure or acute exacerbation of COPD), and 27 were excluded for reasons including pregnancy, coagulation disorders, or patient refusal to provide informed consent. Ultimately, 216 eligible patients were enrolled and allocated into two groups with 108 patients each (Figure 1). The mean age of the cohort was 61.2 ± 9.8 years, and 118 participants were male. No significant differences were observed between the two groups in baseline characteristics, including gender, age, BMI, ASA classification, smoking history, medical history, and type of procedure (all *P* > 0.05), as detailed in Table 1.

**TABLE 1:** Comparison of baseline characteristics between the two groups.

Characteristics	The press-needle group (*n* = 108)	The control group (*n* = 108)	*P*-value
Age, year	61.2 ± 9.8	60.5 ± 10.2	0.595
Gender (male/female)	62 (57.4)/46 (42.6)	56 (51.9)/52 (48.1)	0.412
BMI (kg/m^2^)	23.5 ± 3.2	24.1 ± 3.5	0.156
ASA grade			0.344
I	42 (38.9)	32 (29.6)	–
II	28 (25.9)	34 (31.5)	–
III	38 (35.2)	42 (38.9)	–
Smoking history	45 (41.7)	48 (44.4)	0.673
Medical history			
Hypertension	38 (35.2)	42 (38.9)	0.567
Diabetes mellitus	19 (17.6)	15 (13.9)	0.444
Asthma	23 (21.3)	19 (17.6)	0.479
Chronic obstructive pulmonary disease	12 (11.1)	10 (9.3)	0.648
Type of procedure			
Transbronchial biopsy	65 (60.2)	59 (54.6)	0.392
Laser therapy	28 (25.9)	31 (28.7)	0.635
Bronchial stent placement	15 (13.9)	18 (16.7)	0.556
Brush biopsy	52 (48.1)	49 (45.4)	0.683
Bronchoalveolar lavage	71 (65.7)	68 (63.0)	0.673

BMI, body mass index; ASA, American Society of Anesthesiologists physical status classification.

### Comparison of the rates of nausea and vomiting at different postoperative time intervals between the two groups

Generalized estimating equation analysis demonstrated that both groups experienced a significant reduction in the rates of nausea and vomiting over time (both *P* < 0.001). The press-needle group had significantly lower rates of nausea (OR = 0.417, 95% CI: 0.193–0.900, *P* = 0.026) and vomiting (OR = 0.355, 95% CI: 0.137–0.919, *P* = 0.033) than the control group. There were no significant group-by-time interactions for nausea (*P* = 0.821) or vomiting (*P* = 0.520), suggesting that the preventive effect of press-needle therapy remained relatively stable across different time points for both outcomes (Table 2).

**TABLE 2:** Comparison of the incidence of nausea and vomiting at different postoperative time intervals between the two groups.

Items	Time	The press-needle group (*n* = 108)	The control group (*n* = 108)	*P*	*P* (between-group effect)	*P* (time effect)	*P* (interaction effect)
Nausea	0–6 h	12 (11.11)	25 (23.15)	0.016	0.026	<0.001	0.821
	6–12 h	8 (7.41)	18 (16.67)	0.030			
	12–24 h	5 (4.63)	12 (11.11)	0.073			
	24–48 h	3 (2.78)	8 (7.41)	0.094			
Vomiting	0–6 h	7 (6.48)	18 (16.67)	0.013	0.033	<0.001	0.520
	6–12 h	4 (3.70)	12 (11.11)	0.034			
	12–24 h	2 (1.85)	8 (7.41)	0.045			
	24–48 h	1 (0.93)	5 (4.63)	0.073			

*P* (between-group effect) indicates the overall difference in vomiting incidence between the press-needle and control groups. *P* (time effect) indicates the change in vomiting incidence over time. *P* (interaction effect) indicates whether the treatment effect differs across time points.

### Comparison of the occurrence of nausea and vomiting by severity grade between the two groups

The number of patients experiencing mild, moderate, or severe nausea (*P* < 0.001) and vomiting (*P* < 0.001) was significantly lower in the press-needle group compared to the control group, as presented in Table 3.

**TABLE 3:** Comparison of the occurrence of nausea and vomiting by severity grade between the two groups.

	The press-needle group (*n* = 108)	The control group (*n* = 108)	*P*-value
Nausea			
Mild	15 (13.89)	30 (27.78)	<0.001
Moderate	8 (7.41)	16 (14.81)	
Severe	2 (1.85)	9 (8.33)	
None	83 (76.85)	53 (49.07)	
Vomiting			
Mild	10 (9.26)	22 (20.37)	<0.001
Moderate	5 (4.63)	13 (12.04)	
Severe	1 (0.93)	7 (6.48)	
None	92 (85.19)	66 (61.11)	

### Comparison of the use of rescue antiemetic medication between the two groups

The total number of patients requiring rescue antiemetic medication was significantly lower in the press-needle group than in the control group [7 (6.48%) vs. 22 (20.37%), *P* = 0.004], as shown in Table 4.

**TABLE 4:** Comparison of the use of rescue antiemetic medication between the two groups.

Items	The press-needle group (*n* = 108)	The control group (*n* = 108)	χ^2^	*P* value
Ondansetron	5 (4.63)	15 (13.89)	8.215	0.004
Dexamethasone	3 (2.78)	10 (9.26)		
Total	7 (6.48)	22 (20.37)		

### Comparison of changes in blood pressure levels between the two groups

Regarding blood pressure, both systolic and diastolic pressures were significantly higher in the press-needle group immediately after the procedure compared to the control group (*P* < 0.001). Relative to pre-anesthesia levels, blood pressure decreased significantly immediately postoperatively in both groups but recovered to pre-anesthesia levels by 24 h postoperatively, as detailed in Table 5.

**TABLE 5:** Comparison of blood pressure between the two groups.

Group	The press-needle group (SD)	The control group (SD)	*P* (between-group effect)	*P* (time effect)	*P* (interaction effect)
Systolic pressures (mmHg)					
Before anesthesia (T0)	120.32 ± 10.23	121.12 ± 11.34	0.586	< 0.001	0.001
Immediately after procedure (T1)	115.23 ± 9.87	110.12 ± 10.56	<0.001		
6 h postoperatively (T2)	118.45 ± 10.12	116.34 ± 11.23	0.148		
24 h postoperatively (T3)	120.11 ± 10.34	119.23 ± 10.45	0.534		
Diastolic pressures (mmHg)					
Before anesthesia (T0)	75.45 ± 8.32	76.23 ± 7.89	0.480	<0.001	0.003
Immediately after procedure (T1)	70.34 ± 7.65	65.45 ± 8.90	<0.001		
6 h postoperatively (T2)	73.23 ± 8.11	71.12 ± 9.01	0.072		
24 h postoperatively (T3)	75.12 ± 8.23	74.34 ± 8.56	0.495		

*P* (between-group effect): *P*-value for comparisons between the press-needle and control groups using the two-sample *t*-test. *P* (interaction effect): *P*-value for comparisons between the press-needle and control groups after controlling for repeated measures using the generalized estimating equation (GEE) method. *P* (time effect): *P*-value for testing the time-trend effect within the GEE model.

## Discussion

This study indicates that press-needle therapy is effective in preventing PONV in patients undergoing bronchoscopy under general anesthesia. Although PONV of varying severity occurred in both groups postoperatively, the press-needle group demonstrated significantly lower rates of both nausea and vomiting compared to the control group. Furthermore, the number of patients experiencing mild, moderate, and severe PONV was consistently lower in the press-needle group, indicating a clear advantage. Concurrently, the press-needle group showed a significantly reduced requirement for rescue antiemetic medication, suggesting that this therapy holds promise for decreasing patient reliance on pharmacologic agents and mitigating associated adverse drug reactions in clinical practice.

Postoperative nausea and is a common perioperative complication following bronchoscopy under general anesthesia, posing a significant clinical challenge due to its complex pathogenesis. Current pharmacological prevention and treatment strategies remain suboptimal (18). Key factors contributing to PONV in this context include mechanical stimulation of the airways, enhanced vagal nerve reflexes during bronchoscopy, and the effects of anesthetic agents such as propofol and opioids on the vomiting center and chemoreceptor trigger zone in the medulla oblongata (19). Additionally, the stress response to anesthesia disrupts autonomic nervous system homeostasis, leading to dysfunction of the neuroendocrine axis and dysregulation of inflammatory mediators, further increasing the risk of nausea and vomiting (20). The interplay of these factors renders patients undergoing bronchoscopy a population at high risk for PONV.

In recent years, the value of acupuncture and related acupoint stimulation techniques in managing perioperative symptoms has gained considerable attention. Previous studies have confirmed that acupuncture can effectively prevent and treat PONV through mechanisms such as modulating autonomic nervous function, improving gastrointestinal motility, and suppressing excitation of the vomiting center (21, 22). As an extension of acupuncture, press-needle therapy involves the sustained, low-intensity stimulation of acupoints via micro-needles fixed superficially on the skin. This method offers advantages including minimal invasiveness, ease of application, good patient tolerance, and prolonged stimulation, making it particularly suitable for perioperative use (23).

According to traditional Chinese medicine theory, postoperative nausea and vomiting are often attributed to “rebellious ascent of stomach qi and dysfunction of qi movement” (24). Specific acupoints on the hand and foot meridians, such as Neiguan (PC6), Zusanli (ST36), and Hegu (LI4), are believed to regulate stomach qi, unblock meridians, harmonize the middle energizer, and suppress vomiting, making them crucial points for intervening in postoperative gastrointestinal dysfunction (25–27). Animal studies have shown that stimulation of the Zusanli acupoint in rats can improve gastric dysmotility and reduce the release of emetogenic substances like serotonin, thereby alleviating chemically-induced or anesthesia-related vomiting responses (28). Clinical research has also corroborated that acupuncture or sustained acupoint stimulation can reduce the incidence of PONV, enhance gastrointestinal motility, and improve perioperative patient comfort (29). These findings suggest that acupoint stimulation may regulate the function of the neuro-endocrine-gastrointestinal axis, alleviating the rebellious ascent of stomach qi and functional disorders triggered by surgical and anesthetic stress.

The results demonstrated that the rates of nausea in the press-needle group was significantly lower than that in the control group (*P* < 0.05), whereas no statistically significant difference was observed between the groups from 12 to 48 h. The rates of vomiting in the press-needle group was significantly lower than that in the control group at 0–24 h postoperatively (*P* < 0.05), whereas no statistically significant difference was observed between the groups from 24 to 48 h. The number of patients experiencing mild, moderate, and severe nausea and vomiting was significantly lower in the press-needle group (*P* < 0.05). According to the results, the preventive effect of press-needle therapy on nausea was primarily observed within 0–12 h postoperatively, whereas its effect on vomiting persisted up to 24 h postoperatively, suggesting that press-needle may exert a more sustained regulatory effect on the vomiting reflex than on nausea. Although no statistically significant differences were observed between the two groups during the 12–48 h period for nausea and the 24–48 h period for vomiting, the numbers of patients experiencing mild, moderate, and severe nausea and vomiting were significantly lower in the press-needle group, indicating that this therapy effectively reduces the severity of PONV. These results suggest that while press-needle therapy is mainly effective in the early postoperative phase, its advantage in alleviating symptom severity holds important clinical value. Future studies may consider increasing the stimulation frequency or combining press-needle with other interventions at 12–24 h postoperatively to further extend the therapeutic window. In the pressure needle therapy group, the need for emergency antiemetics was significantly reduced (*P* < 0.001). In particular, the use of Ondansetron and dexamethasone decreased. These results suggest that pressure needle therapy reduces dependence on drug antiemetics and reduces the risk of associated adverse effects. This is particularly important for patients who cannot tolerate these drugs or who have contraindications. In addition, the press-needle therapy group had higher postoperative blood pressure values compared to the control group (*p* < 0.001), suggesting that the therapy contributes to the stabilization of perioperative hemodynamics by regulating autonomic nervous system function.

Acupoint stimulation therapy is non-pharmacological therapy that includes transcutaneous electrical acupoint stimulation (TEAS), acupuncture, auricular acupuncture, acupressure, press-needle therapy, electroacupuncture (16, 30, 31). A growing number of randomized controlled trials (RCTs) have shown that acupoint stimulation alone or in combination with antiemetic drugs can reduce the incidence of PONV. The network meta-analysis of RCTs revealed that compared with the control (sham acupoint stimulation or blank control), antiemetic alone could significantly reduce the incidence of postoperative vomiting (POV) (RR 0.49, 95% CI: 0.36–0.69), but could not significantly reduce the incidence of PONV and postoperative nausea (PON) (RR 0.49, 95% CI: 0.36–0.69; RR 0.81, 95% CI: 0.59–1.10; respectively); both TEAS and electroacupuncture alone significantly reduced the incidence of PONV, PON, and POV, and combined with antiemetic was usually more effective than single acupoint stimulation (32). In addition to acupoint stimulation therapy, many antiemetic drugs are available for prophylaxis. They have various mechanisms of action and side effects, but there is still uncertainty about which drugs are most effective with the fewest side effects. Two network meta-analyses showed that at least seven single drugs have a prophylactic effect on postoperative vomiting, and no further efficacy studies are needed (32–34). However, more research is still required to investigate their potential adverse reactions. Therefore, compared with pharmacotherapy, acupuncture may offer superior effects with fewer adverse reactions. Further research is needed to compare the preventive and therapeutic effects of acupuncture and antiemetic drugs on postoperative nausea and vomiting after general anesthesia.

We recognize that other stimulation methods (electroacupuncture, TEAS, acupressure) have a stronger evidence base for PONV prevention. We do not claim press-needle is superior; rather, we present it as a practical alternative with specific logistical benefits for bronchoscopy under general anesthesia. According to the 2025 Cochrane network meta-analysis, all PC6 stimulation modalities-invasive or non-invasive-produce comparable clinical effects, as no significant subgroup differences were found for any primary outcome (35). Given this efficacy equivalence, technique choice depends on contextual factors. For bronchoscopy-characterized by short procedures (20–30 min), total intravenous anesthesia, and uninterrupted recovery-press-needle offers four advantages: (1) single application provides 48-hour sustained stimulation; (2) no electrical equipment or repeated interventions; (3) minimal training for placement; and (4) good patient tolerance (23).

This study has several limitations. First, the sample size was relatively small. Second, the follow-up period was short (48 h), and the optimal acupoint selection and needle retention time for press-needle therapy were not thoroughly investigated. Third, this study employed a quasi-experimental design with sequential allocation based on enrollment time. This non-randomized design introduces potential sources of bias. Fourth, no sham-needle control was included, which may have introduced placebo effects and compromised the ability to attribute the observed effects solely to the press-needle intervention. Fifth, since all enrolled patients were of Chinese origin, this may limit the generalizability of the results to other regions or ethnic populations. Sixth, the WHO severity grading system used in this study is more commonly applied in CINV research than in PONV assessment. The lack of a validated PONV-specific instrument therefore represents a limitation. This study is a quasi-experimental study, using a non-randomized historical control design, according to the Oxford Center for Evidence-Based Medicine evidence level classification, the evidence level is 4; according to the GRADE classification system, the quality of evidence is low to extremely low. Therefore, the conclusions of this study should be interpreted with caution, and large-sample, multicenter, sham-controlled randomized controlled trials are warranted to further validate the clinical efficacy of press-needle therapy in this patient population.

In conclusion, this study demonstrates that press-needle therapy can effectively prevent the occurrence of PONV following bronchoscopy under general anesthesia, reduce the severity of symptoms, decrease the need for rescue antiemetics, and has a minimal impact on vital signs. It offers advantages of safety, efficacy, and ease of operation.

## Data Availability

The original contributions presented in this study are included in the article/supplementary material, further inquiries can be directed to the corresponding author.
